# What is it like to microdose LSD for depression? a thematic analysis of participant interviews from an open-label trial

**DOI:** 10.1177/20451253251396253

**Published:** 2025-12-04

**Authors:** Carina Joy Donegan, Dimitri Daldegan-Bueno, Rachael L. Sumner, Anna Forsyth, Will Evans, Nicholas R. Hoeh, Frederick Sundram, David Menkes, Suresh Muthukumaraswamy, Lisa Reynolds

**Affiliations:** Department of Psychological Medicine, University of Auckland, 85 Park Rd, Auckland 1023, New Zealand; School of Pharmacy, University of Auckland, Auckland, New Zealand; School of Pharmacy, University of Auckland, Auckland, New Zealand; School of Pharmacy, University of Auckland, Auckland, New Zealand; Mana Health, Parnell, Auckland, New Zealand; Department of Psychological Medicine, University of Auckland, Auckland, New Zealand; Department of Psychological Medicine, University of Auckland, Auckland, New Zealand; Department of Psychological Medicine, University of Auckland, Auckland, New Zealand; School of Pharmacy, University of Auckland, Auckland, New Zealand; Department of Psychological Medicine, University of Auckland, Auckland, New Zealand

**Keywords:** lysergic acid diethylamide, major depressive disorder, microdosing, open-label, psychedelics, qualitative research

## Abstract

**Background:**

Depressive disorders affect approximately 280 million globally, with many finding treatments ineffective or limited by side effects. Growing evidence suggests that psychedelic therapies may help alleviate depressive symptoms. Among these, lysergic acid diethylamide (LSD) microdosing shows promise for major depressive disorder (MDD). However, research on LSD microdosing in clinical populations remains limited.

**Objectives:**

This study aimed to understand the experiences of individuals participating in an open-label trial of LSD microdosing for MDD.

**Design:**

Open-label pilot trial in target population (MDD; phase IIa).

**Methods:**

Seventeen participants with MDD completed an 8-week LSD microdosing regimen, dosing twice weekly. Following the intervention, participants underwent semi-structured interviews regarding their experiences. Data were analysed using thematic analysis.

**Results:**

Themes were grouped into five categories: enhanced self-determination, increased connectedness, improved cognitive processing, better emotional well-being, and negative effects.

**Conclusion:**

Reported effects appeared to reinforce one another; that is, self-determination led to feeling more connected, which enhanced cognitive processing and ultimately improved emotional well-being and reduced depressive symptoms. However, this effect was not universal; some individuals reported negative effects or no significant improvement from microdosing LSD. This variability may be due to individual differences in response, insufficient dosage, or the treatment’s lack of effectiveness for some individuals. The presence of side effects highlights the need for a careful titration protocol, while the lack of symptom improvement in some cases reinforces that microdosing is not a guaranteed solution, and expectations should remain realistic. The absence of a placebo control represents a key limitation as it precludes attribution of observed changes specifically to LSD.

**Trial registration:**

ANZCTR, ACTRN12623000486628. Registered on 12 May 2023 (https://www.anzctr.org.au/Trial/Registration/TrialReview.aspx?id=385758).

## Plain language summary

Depression affects millions of people worldwide, and many find that current treatments either don’t work or have unwanted side effects. Recent research suggests that psychedelic substances, like LSD, may help improve mood when used carefully small amounts. This practice is known as LSD microdosing. Despite growing interest, there is very little controlled research on how LSD microdosing affects people with depression.

In this study, we invited 17 adults with depression to take very low doses of LSD twice a week for eight weeks. After the study, we asked them about their experiences to understand how microdosing affected them. Participants reported a range of experiences. Many described feeling more motivated to engage in daily activities, a stronger sense of connection with others, clearer thinking, new personal insights, and overall improvements in emotional well-being. The improvements participants described often seemed to build on each other—for example, feeling more connected encouraged them to take part in more activities, which then helped them feel mentally clearer and emotionally better.

However, not everyone benefited. Some participants reported negative experiences or no noticeable improvement, suggesting that microdosing may not work for everyone. The study also did not include a placebo comparison, so it is unclear whether the changes were due specifically to LSD.

Overall, these findings suggest that LSD microdosing may offer some people with depression new ways to feel more connected, motivated, and emotionally balanced. At the same time, careful monitoring is important due to potential side effects, and expectations should remain realistic.

## Introduction

Recent years have seen growing interest in microdosing psychedelics,^1^ which involves taking repeated, very small doses of psychedelic substances over weeks or months.^2^ Microdosing typically focuses on classic psychedelics (5HT-2A receptor agonists) such as lysergic acid diethylamide (LSD), *N,N*-dimethyltryptamine (DMT), and psilocybin. This renewed psychedelic interest is linked to exploring their therapeutic potential in mental health.^3–8^ However, rigorous research in clinical populations is limited, with the exception of one study in individuals with Attention-deficit/hyperactivity disorder (ADHD), which found no benefits over placebo.^9^

Major depressive disorder (MDD) is a clinical condition that appears well-suited to microdosing research. MDD not only profoundly impacts the individual, their family, and society,^10–12^ it is also highly prevalent, affecting over 260 million individuals worldwide.^13^ Despite the substantial burden of depression, current pharmacological antidepressant treatments are insufficient, limited by slow onset, variable tolerability, detrimental side effects, and partial or total lack of efficacy in approximately one-third of patients.^14–16^ In light of these concerns, exploring novel therapeutic approaches could be highly impactful. LSD microdosing is promising in this context, with its growing underground use^17^ suggesting potential mood,^2,18,19^ cognition and energy improvements.^16,20–22^ Community users also describe stronger social connections, enhanced sexual experiences, and reduced depression and anxiety.^2,6,17,19,23,24^ Retrospective surveys of community microdosers indicate that mental health improvements are both a key motivation and a primary reported outcome of microdosing,^17–19,23,25^ with depression being the most common indication.^6^ Despite problems with clear selection bias, one online study of self-directed microdosers found that 90% rated it as helpful for their mental health condition, compared to only 35.5% for antidepressants.^26^ Tracking of community microdosers using validated subjective measures has also shown decreases in depression and anxiety, along with improvements in mental well-being over 4 weeks^23^ and significant reductions in depression and stress symptoms over 7 weeks.^6^ Thus, depression is a natural indication for study. However, some clinical studies in healthy volunteers shed uncertainty about the benefits of microdosing, with reports of either no clinically significant changes^27,28^ or only modest transient improvements.^29^ While LSD microdosing appears promising, its effects can vary. This uncertainty highlights the need for further rigorous clinical investigation in MDD.

To conduct rigorous trials where findings are truly understood, qualitative inquiry is an essential, yet often-overlooked, component. Qualitative interviews add significant value by offering understanding above and beyond standardised assessments, uncovering unforeseen experiences, and amplifying participant voice.^30–32^ In the context of microdosing for MDD, understanding participants’ lived experiences in a clinical setting is particularly important, as the effects of psychedelics can vary widely, and MDD itself is highly complex and heterogeneous.^33^ Predetermined quantitative methods may be less effective in capturing these individualised experiences. Despite the growing interest in microdosing, qualitative research within clinical trials for psychiatric disorders remains limited. This gap highlights the need for further qualitative research to deepen our understanding of this area and prioritise what participants find meaningful rather than relying solely on researcher-selected measures.^34^ Elaboration through qualitative work is key to understanding the unique phenomenon of psychedelic microdosing in MDD.^35^

The present study explored the experiences of participants with MDD who completed a clinical trial of LSD microdosing. Post-treatment regimen semi-structured interviews were completed, allowing for a thorough examination of participant experiences.

## Methods

### Procedure

This report provides a secondary analysis of an open-label pilot study examining LSD microdosing in individuals with MDD. A full methods protocol was published prospectively in Donegan et al.^36^ All data were collected from July 2023 to April 2024. Seventeen participants with MDD microdosed twice weekly for 8 weeks (16 doses total), with 15 taken at home and one in the clinic. Doses started at 8 µg and were titrated between 4 and 20 µg based on subjective experience. Participants were instructed to increase their dose if they felt little or no effects and to reduce it if they experienced any disruptions to their regular activities/daily functioning. On dosing days, participants were encouraged to engage in a self-selected psychologically beneficial activity an hour after dosing. This could be anything that felt it would be helpful to the participant, that is, getting a coffee, going for a walk, meditation, etc. After completing the 8-week regimen, participants took part in a semi-structured interview designed by researcher CJD, based on a prior LSD microdosing trial^29^ (for the interview schedule, see the Supplemental Material). Researchers CJD, DDB and SM conducted interviews during the final trial visit at the Auckland Clinical Research Centre, lasting 20–60 min depending on how much participants talked about their experience. All participants provided written informed consent, allowing the research team to transcribe and analyse their interview data. The reporting of this study conforms to the Standards for Reporting Qualitative Research (SRQR) statement^37^ (see Supplemental Materials for checklist).

### Thematic analysis

Thematic analysis (TA) was selected because of its flexibility in generating themes from the data, which do not need to be bound to a specific theory.^38–40^ Inductive TA also allowed iterative processing between CJD, LR and SM and a reflexive approach ensured awareness of bias and prioritisation of participant response. Interviews were recorded and transcribed verbatim using otter.ai with human oversight to ensure accuracy. Transcripts were read through twice by CJD to ensure clarity and familiarity with the data, as per Braun and Clarke.^40^ All participant quotes were then anonymised, and filter utterances removed. CJD then generated initial codes inductively from the data using NVivo. Eighty-four codes were generated. After consultation with LR and SM, these were narrowed to 16 codes. Coded transcripts were then examined for themes relating to LSD microdosing experience and any changes or lack thereof reported over the trial. These were then defined and named.

### Researcher declaration

This study adopts a critical realist approach, recognising the existence of an objective reality while also considering participants’ and researchers’ subjective interpretations. The findings were reviewed and developed by a multidisciplinary team, including experts from psychological medicine, health psychology, pharmacy, and population health, as well as clinicians such as psychiatrists, psychologists, and physicians. Although this diverse expertise enriches the analysis, the shared emphasis on health and medicine may introduce bias that prioritises health-related perspectives.

## Results

### Demographics

Seventeen participants completed the 8-week regimen of microdosing and undertook a semi-structured interview. The sample included four females and 13 males aged between 24 and 60 years. The majority identified as New Zealand European (*N* = 13). Additional demographics are given in Table 1.

**Table 1.** Demographic characteristics of participants undertaking a semi-structured interview after an 8-week LSD microdosing regimen.

Characteristic	Mean (SD)
Age in years	39.67 (11.82)
	*n* (%)
Gender	
Female	4 (23.53)
Male	13 (76.47)
Psychedelic use	
Prior psychedelic use	10 (58.82
Psychedelic naïve	7 (41.18)
Highest qualification	
Tertiary or other diploma	4 (23.53)
Undergraduate	9 (52.94)
Postgraduate	4 (23.53)
Race/Ethnicity*	
European	13 (76.47)
Ma¯ori	2 (11.76)
Asian	2 (11.76)
Pacific Peoples	2 (11.76)
Current antidepressant use	
Yes	3 (17.65)
No	14 (82.35)

* The race/ethnicity *n* = 19, as some individuals identified with more than one ethnic identity.

LSD, lysergic acid diethylamide.

### Thematic analysis

Five themes were identified, including (1) enhanced self-determination, (2) increased connectedness, (3) improved cognitive processing, (4) better emotional well-being, and (5) negative effects. These themes and associated subthemes are presented in Table 2.

**Table 2.** Themes and subthemes of individuals with MDD interviewed after an 8-week regimen of LSD microdosing.

Theme	Subthemes
Enhanced self-determination	• Motivation to do activities • Mental/cognitive motivation • Doing activities makes a difference
Increased connectedness	• Connection to Self • Connection to others/social motivation • Connection to Something bigger – world/nature/spirituality
Improved cognitive processing	• Better emotional processing • Increased focus
Better emotional well-being	• Improved mood • Reduced depressive symptoms
Negative effects	• Side effects • No symptom change

LSD, lysergic acid diethylamide; MDD, major depressive disorder.

#### Enhanced self-determination

When asked about their experience, many participants reported concepts related to self-determination. This involved a general sense of agency and control in their life in contrast to the passivity of their depression. Self-determination reflected an enhanced capacity to make deliberate choices, pursue meaningful activities and actively shape their recovery.^41^ It fell into three areas: (1) motivation to do activities, (2) mental/cognitive motivation and (3) doing activities makes a difference. This boost, often attributed to the doses themselves, facilitated engagement with necessary, desired or beneficial activities.

##### Motivation to do activities

Many participants described a renewed drive for activities, akin to pre-depression levels: ‘*My friends and my partner have noticed that I want to do things, when I didn’t want to do anything before*’ [^#^14]. Another participant similarly expressed that ‘*I liked being out and about. It always gave me a good [feeling of] let’s go somewhere. Let’s do something*’ [^#^17]. For some, this motivation was linked to energy and new feelings of capability ‘*I’ve struggled getting out of bed for years. . . but now I have energy as soon as I wake up and it feels sustained*’ [^#^12] and, for others, this motivation was linked to their increased physical activity ‘*I have been doing a whole lot more exercise. And with the exercise and the running and going to the cricket nets, I’ve been able to do more because of that motivation*’ [^#^2]. These reinforced feelings of being competent and capable. Some participants also reported more enjoyment in activities on dosing days: ‘*On the dosing days, if I did something. . .I was more eager to do that activity. And I’d enjoy it a little bit more*’ [^#^14].

##### Mental/cognitive motivation

Feelings of self-determination were also reported as related to motivation in mental and cognitive domains. These were linked to feelings of capability, where participants reported a boost in mental motivation, making it easier to tackle tasks that would have otherwise felt overwhelming, one individual noting they felt ‘*Good motivation, good productivity*’ [^#^17] and another noting ‘*I’m more willing to and more wanting to try new things*’ [^#^14]. Participants also reported a drive to engage with mentally difficult tasks:

*I guess it’s just easier to get up and do things. I procrastinate a lot. And if something doesn’t really need to be done, I can put it off and off. But I definitely felt there were sometimes I actually wanted to do it’* [^#^13].

Others noted improved motivation with creative tasks. During depressive periods, many had viewed creativity as a waste of time due to low capacity. For one participant, this changed after microdosing:

*‘As a quite driven person that likes to get shit done, being creative seems really superfluous, it has no real point, but I think recently I’ve realised that it can just be for me, I think it’s actually really good for my soul’* [^#^1].

Another participant also highlighted this: ‘*It’s just given me a whole lot more motivation, I started DJ’ing again after 20 odd years*’ [^#^15].

##### Doing activities makes a difference

In addition to motivation to do activities, participants also noted that the act of doing activities in themselves made a large difference to their overall mental well-being. They seemed to enjoy being active again and the engagement that it brought. For instance, one participant described they ‘*felt just generally better, in my body from going out and doing those things, and not sitting at home, wallowing in the sadness and ruminating*’ [^#^14]. Another also noted heightened satisfaction with taking part in activities, ‘*I’ve been going and doing things a lot more with my daughters, whereas before the trial, I wasn’t really going out with them*’ [^#^2].

One participant noted this effect for both physical and mental activities: ‘*I’ve been able to maintain my activity, and physical health which has been great, and I think that’s had flow on effects to my mental health*’ and ‘*I’ve reconnected with that [creativity] and it’s quite meditative for me which I really enjoy*’ [^#^1]. For some, the combination of the trial and trying to do activities made the difference: ‘*I felt generally more positive, less likely to feel tunnel vision, especially on negative things. I think it’s probably the trial in combination with making an effort*’ [^#^14]. Here, it seems that microdosing might provide self-determination; however, inclusion of psychologically beneficial activities in the treatment protocol can further enhance positive effects.

#### Increased connectedness

During the trial, several participants reported profound connectedness across domains of the self, others, and the world around them. This connectedness manifested as increased appreciation for the world, relationships, and themselves.

##### Connection to self

Many participants reported a sincere connection to themselves throughout the trial; this was often linked to a sense of understanding surrounding their depression. One participant described that ‘*it’s helped me personally to get a better understanding of, and be more able to engage with, my mental health*’ [^#^6]. Others linked this self-connection to acute awareness of their moods and ability to monitor these: ‘*I would be more aware of how I was feeling and would feel sometimes, slightly more anxious or irritable, and things like that. So yeah, I think it was really helpful because it made me more aware of how I was feeling*’ [^#^14].

Others reported deep exploration of their own minds and sitting with their experience: ‘*It made me like, look a bit deeper. Especially on the dose days, I feel like I can really get into my mind*’ [^#^12]. For some, this connection felt positive and prompted acceptance of themselves: ‘*and I feel a bit more sort of comfortable in myself*’ [^#^16].

##### Connection to others

Participants also reported increased connectedness to others: ‘*The connection I have felt with all things has been fantastic. I caught up and reconnected with some old friends that I haven’t spoken to, and I’ve just been connecting*’ [^#^15]. Some were surprised by their ability to engage with others and the closeness it fostered, with one participant expressing: ‘*I’ve been catching up and doing all these things that I don’t usually do. And I think it’s been a real positive.*’ [^#^2].

For some, the connectedness to others was tied to a broader desire to re-engage with the world, something they may have struggled with in the throes of their depression: ‘*Before, I would have been very happy to not leave the house and not talk to anyone. But now, I want to get out there and do things and see people and meet people*’ [^#^12]. These participants described feeling closer to others and that their time spent connecting was more enjoyable: ‘*I really wasn’t present with the family. And I felt a bit more connection there, a bit more presence, fun*’ [^#^16]. This increased connection was also linked to a motivation to engage socially. For example, one person explained that ‘*before taking the drug I found it hard to start conversations. . . but these days, it’s very easy*’ [^#^2]. Another participant described their feelings as akin to those before depression:

*‘I did a dosing day, and I went for a walk to these shops, and I was talking to people, talking to shopkeepers and talking to strangers. And that’s what I used to do when I was going out before depression sort of sunk into my life’* [^#^15].

Some participants also reported reduced social anxiety, making interactions easier: ‘*During the trial, I have been really social and felt a lot less social anxiety. I’m less critical of myself among people*’ [^#^5]. The enhanced social engagement also seemed to play a role in the mental health improvements reported by participants:

*‘I still have a limit to my social battery. But I want to see people and I want to socialise, and I find it fulfilling and enjoyable for the most part…I’m in a place where I can enjoy talking to other people, and I’m not so focused on the negative things I’m feeling or thinking’* [^#^14].

##### Connection to something bigger than themselves (world/nature/spirituality)

Connectedness was also described in a deeper closeness to nature and the world, feeling connected to something bigger than themselves. One participant reflected on their symptom reduction in relation to their connection to nature:

*‘It’s the connection with nature. I went for a swim in the ocean on a dosing day and just felt the saltwater on my body. It was just, oh my god, this is so cool. I also took a walk through the bush, listening to the birds, and it really brought me out of my depressed shell’* [^#^15].

Such connections to nature and the world also extended out to feeling a stronger bond to animals, with one participant remarking: ‘*I felt really drawn to nature, spending really good quality time with my animals. So, I would just lie on the floor [laughs], and I had a real bond with the cat*’ [^#^4]. Connection to the world was related to a drive for life in some. Participant ^#^17 shared ‘*[I’m] feeling a bit more purpose and like there’s a place for me in the world*’. Participants also reported connection prompting a profound sense of belonging to the world:

*‘I definitely feel more a part of the world, which is great. I used to look at the world and people very negatively. I didn’t want to meet anyone or talk to anyone… But now it’s so beautiful. And it’s a great thing about the world. I just feel more ready for that connected feeling. It’s just everywhere. I’m just drooling in the fact; I see more beauty in the world now, as well as feeling connectedness. I noticed now there’s constantly the chance to be feeling good and be looking at something beautiful, appreciating something that’s way easier to find now, it’s just everywhere’* [^#^12].

Some participants also reported a deepened spiritual connection. One participant reported a connection to their Buddhist practices: ‘*I find on dosing days I am a lot more connected to the Gohonzon and chanting, I feel a lot more inner peace* [^#^15]. Another linked their connection with nature and spirituality:

*‘I think we’re spiritual beings having a human experience, and the human experience is the hard part, but the spirituality is deep within us… I think feelings spirituality; there’s definitely a connection there to sit and just look at a blade of grass and just see the beauty in it and actually see the nature in it. And the fact that man, what an unbelievable planet, and the stuff that happens, and I’m starting to realise I’m part of it’* [^#^4].

And one spoke of the improvements they felt were due to ‘*an amalgamation of all of those things, the LSD, the spirituality and mindfulness, yeah*’ [^#^1]. Another described a revelation related to being part of the broader picture, not just existing in their mind: ‘*We’re the same if we listen, I mean really listen, not to this thing but listen to the broader picture*’ [^#^4].

#### Enhanced cognitive processing

Several participants described experiencing enhanced cognitive processing, including mental clarity and insight, emphasising their ability to think more clearly, stay motivated, and look at the bigger picture. They reported an improved capacity to cope with and process emotions and also a better ability to maintain focus.

##### Better emotional processing

Participants in this trial described changes in their ability to process their emotions. For one participant, this was important in putting things in perspective, specifically the ability to see the bigger picture, rather than dwelling on internal strife. They indicated that the microdosing ‘*certainly allowed me to stop focusing on little details, or getting stuck on parts of the problem, but rather, it let me zoom out and go, okay, what’s actually the bigger picture?*’ [^#^6].

As might be expected, participants continued to encounter challenging life events during the trial; however, they reported that microdosing enabled them to better cope. For instance, one participant shared:

*‘That incident, that would have affected me. It did affect me deeply. But it would’ve taken me down, I would have felt depressed, I would have closed off…whereas, yesterday, I was able to have a cry about it, talk about it, talk to people, connect with people… it was quite different. How I react to a tragedy and a major event like that is quite different’* [^#^15].

Another participant also described LSD microdosing as a turning point in coping with a negative life circumstance. *For me, it’s been a game-changer. I’m going through some really tough stuff at the moment, and I’m not phased. It’s not as important* [^#^4]. Others also reported an enhanced ability to cope with longstanding emotional challenges:

*‘The past month has been the one-year anniversary of my mum dying, and a lot of things going on. And I think if I wasn’t on this trial, I wouldn’t have coped nearly as well’* [^#^12].

Participants also reported increases in mindfulness and ‘now presence’, which helped them feel less numb to the world. One participant described ‘*I thought about things a little bit more and was more aware of the present than just going through the day and not really feeling a whole lot*’ [^#^13], and another noted ‘*I seem to be able to take things at face value. Live in the moment and get on with it. Without panicking and freaking out. . .well, being present*’ [^#^4]. This change in perspective did not always improve mood, but it did enhance emotional awareness, with one participant reporting, ‘I *still get emotional, but not as wrapped up in my emotions. I’m able to sit with them and not let it take over me*’ [^#^12]. Similarly, another participant stated, ‘*I feel emotions quite strongly, but I have recently been able to create space around those emotions and not be absorbed by them*’ [^#^1].

##### Increased focus

Some participants discussed increased focus after taking the LSD. One described this as feeling a ‘*mental sharpness*’ [^#^10], and another described they could ‘*really zone in overall*’ [^#^13]. This focus was reported to increase their ability to engage with work and get things done, as reported by one participant, ‘*I could concentrate on something for longer and not get so distracted, I just could get into things more*’ [^#^14]. Increases in concentration for some also seemed to be linked to feelings of awareness or intensified sensations, as noted by one participant, ‘*I was focusing on things that I might not normally focus on. . ., senses just felt a bit heightened, I guess*’ [^#^13]. This was also reported as lending itself to a general feeling of intelligence as demonstrated by one individual, in reference to playing the word game, WORDLE: ‘*I felt a bit smarter. . . the words would just pop up into my head very quick*’ [^#^7].

#### Increased emotional well-being

Over the trial, many participants reported general improvements in their emotional well-being, including improved mood and a reduction in depressive symptoms. This was generally reported as an outcome of other improvements over the trial.

##### Improved mood

Throughout the trial, participants reported they felt in better moods. One participant noted they were ‘*just real happy, just in a good mood*’ [^#^17]. Another expressed thanks for the mood effects, ‘*Grateful for the opportunity and the positive effects I’ve felt. . . I’m generally really, really, really happy*’ [^#^14]. Yet another participant noticed: ‘[its] *definitely life changing. I feel like this will carry on with me for a really long time*’ and described their change in mental health as ‘*very affirming. It’s made me feel better in every aspect of the world, who I am, my mental health, and everything*’ [^#^12]. Others reported more aggregate changes: ‘*It’s had a cumulative effect. So, as I’ve gone on, the gaps between dosing have been cruisier, it feels like it’s sort of built in my system*’ [^#^4].

Some participants reported feeling better moods than on antidepressants: ‘*On those SSRI antidepressant drugs, you can feel numb*’ and on LSD ‘, *this allows you to feel those peaks and troughs and the troughs aren’t too bad*’ [^#^15]. Some participants experienced such significant improvements that they considered stopping antidepressants: ‘*I feel less depressed in general, which is great. The idea of potentially coming off antidepressants seems more feasible and possible*’ [^#^12]. This was echoed by another participant who stated, ‘*This has been the most effective thing I’ve tried*’ [^#^14].

##### Reduced depressive symptoms

Participants also reported a reduction in depressive symptoms over the trial. One individual noted they ‘*just feel less sad about everything now. It’s a beautiful time to experience emotions and be with people. And I don’t know, for some reason, it just feels a bit easier*’ [^#^12]. Another participant described a dulling of depressive symptoms: ‘*I feel it potentially has actually dumbed down or helped remove some of that sort of negative cloud that would generally be sitting over me*’ [^#^9]. Some noted they felt that microdosing helped prevent them from slipping into a more depressed state: ‘*I only had one week during the trial, where I felt as though I could have slipped into a really quite significant depressive period. I ended up not slipping into that*’ [^#^5]. These individuals reported that LSD could not take away the pain of living; however, it made it more bearable:

*‘I do feel like I’ve had less periods where I’ve been down. And the feeling I get is not so much any euphoria, or general happiness, it’s more like, I just feel a bit more lightheaded and carefree’* [^#^16].

#### Negative effects

A key theme that participants talked about was negative effects, which included both having no noticeable impact and the presence of unwanted side effects. Even among those who reported improvements, some still experienced such side effects.

##### No change

Some participants reported LSD left them feeling unchanged: ‘*To begin with, I wanted to feel something, but I’m pretty sure for one reason or another, it didn’t impact me – positively, negatively, or in between*’ [^#^8]. Some participants described their experiences with microdosing as incomparable to being on antidepressants: ‘*yeah, it’s not an antidepressant. I’ll say that much*’ [^#^6]. Another participant experienced so little difference that they stated they ‘*would believe you if you told me that I was on placebo*’ [^#^11]. Some participants said this was noticeable to others: ‘*I guess my partner would be the best person to notice, and she didn’t really. She’s made a couple of comments that she doesn’t think anything’s really changed*’ [^#^13].

##### Unwanted side effects

As with many medications, some participants noted adverse side effects. One was sleep disruptions, with one participant reporting ‘*on dosing days, trying to go to sleep is a little more difficult*’ [^#^4] and another noting ‘*it was easy to get to sleep the next night* [after dosing] . . . *because I was so exhausted from the previous night not being able to get to sleep*’ [^#^2]. Some participants, however, reported more persistent issues: ‘*My sleep hasn’t been as good. Especially over the last four to six weeks*’ [^#^15]. Other participants noted unwanted mental effects: ‘*My partner said I’d be worse the day after the dosing day, or more tired or more depressed. Not to the extent of a full depression*’ [^#^6] and ‘*I’ve generally got it* [anxiety] *anyway. But I was getting anxiety at home, because I was generally dosing at home, and I don’t generally get anxiety at home*.’ [^#^11].

Others noted more physical side effects such as: ‘*a bit of feeling spaced out or dizziness on some doses*’ [13] and ‘*light-headedness, and slight dizziness*’ [16]. For some, this also occurred on the day after dosing in a form akin to a hangover: ‘*I do get a bit of a hangover the next day. . . but it’s not depressing. It’s just physically I feel a bit sluggish and a bit slow*’ [^#^4]. These side effects occurred in people who experienced little improvement with LSD and those who showed drastic improvements.

#### Proposed mechanism of LSD microdosing in depression

**Figure 1.**
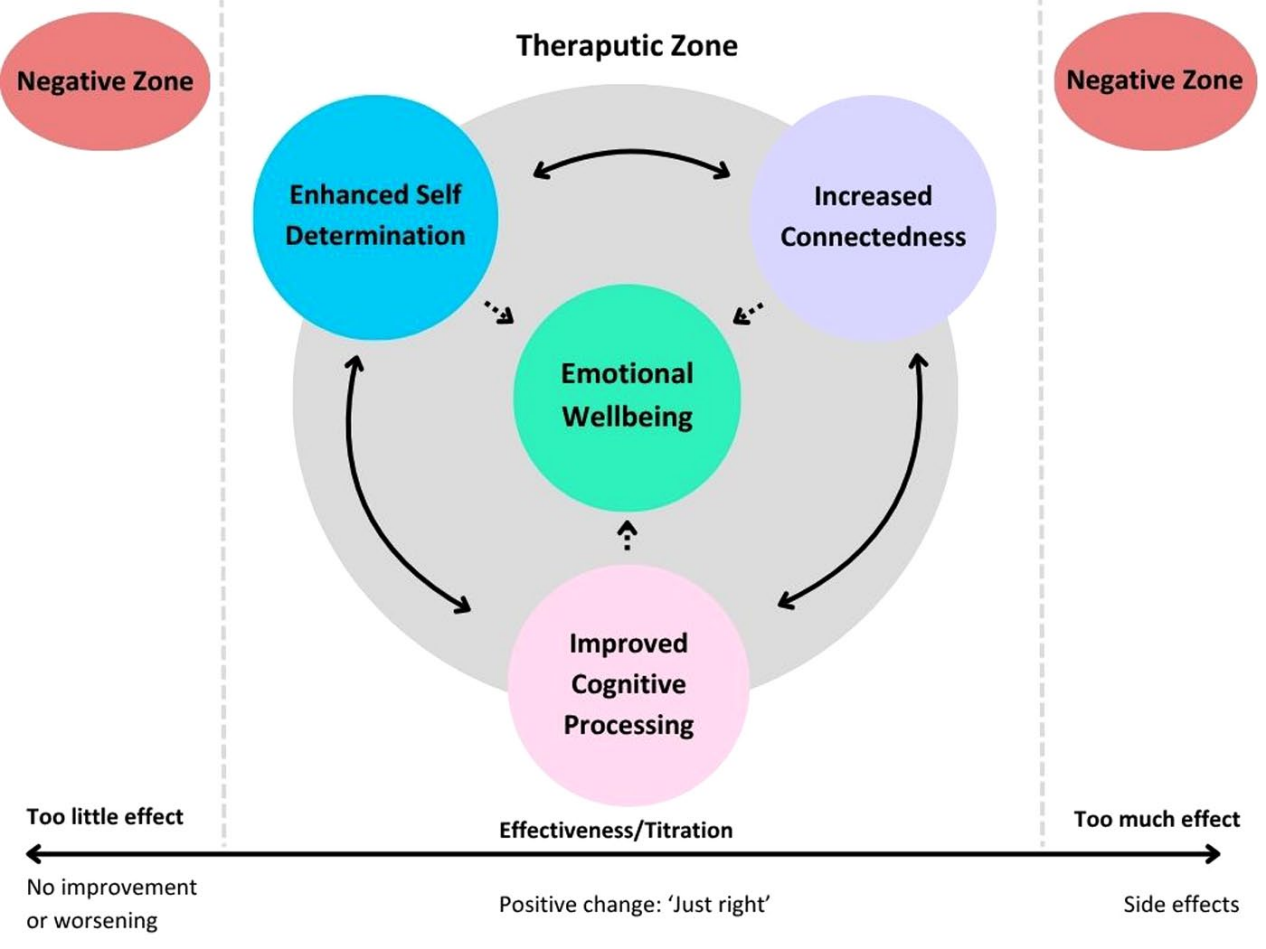
Proposed cyclical therapeutic mechanism for lysergic acid diethylamide microdosing in depression with titration scale.

We propose a cyclical mechanism through which LSD microdosing may confer benefits to individuals with depression (see Figure 1). Our participants reported three main improvements: enhanced self-determination, increased connectedness, and improved cognitive processing, which appeared to interact with one another to enhance emotional well-being. Self-determination referred to increased motivation to engage in daily activities, with participants often noting that doing these activities made them feel better. Connectedness involved a stronger sense of connection to themselves, to others, and to the world. Enhanced cognitive processing encompassed greater mental clarity, improved focus, and the ability to cope with experiences, often leading to new insights. Emotional well-being was the resultant outcome, in turn, reflecting reduced depressive symptoms.

For example, participants reported that their increased engagement in activities and motivation (self-determination) led to more interaction with their environment, their loved ones, and engagement in activities that aligned with their identity. This, in turn, fostered a deeper sense of connection to the world, others, and themselves. This connection and active engagement in life created a sense of purpose and accomplishment, which facilitated individuals to view situations with a clearer perspective, ultimately leading to enhanced cognitive processing both emotionally and in terms of focus. This cycle seems to be multi-directional, as shown in Figure 1, forming a positive feedback loop where each aspect enhances the others. In other words, we propose that increased self-determination, connectedness, and cognitive processing help individuals feel better, leading to a reduction in depressive symptoms and stronger mental health.

We propose that these improvements occur within the ‘therapeutic zone’, where individuals experience the beneficial effects of LSD microdosing. Ideally, this represents the therapeutic range targeted by the titration protocol. However, if a person has too little or too much of a dose, they might move into the ‘negative zones’ where challenges can arise. Figure 1 shows how a dose that is too little (left side of scale) might not result in meaningful symptom changes. This could stem from one of two factors: either microdosing is ineffective for the individual, or they have not yet reached an optimal dose through titration. Furthermore, a dose that is too high (right end of the scale) may lead to adverse outcomes such as anxiety or racing thoughts. Thus, titration poses a pragmatic solution to tailor dosing to individual needs so that different people can find the dose that keeps them in the therapeutic zone.

It is important to acknowledge here the heterogeneous nature of MDD^33^ and the possibility that some individuals may not respond to microdosing at all, regardless of dose.

## Discussion

LSD microdosing shows therapeutic potential for MDD, with non-clinical reports suggesting benefits. Given the high prevalence and widespread impact of MDD, it is crucial to study it thoroughly. Qualitative research is particularly valuable in capturing subjective experiences often missed by standard assessments. In the current study, several themes were identified through TA of participants’ accounts of their LSD microdosing experience, including: (1) enhanced self-determination, (2) increased connection, (3) improved cognitive processing, (4) better emotional well-being, and (5) negative effects. While these results cannot be directly attributed to LSD microdosing, as no placebo control was used. They nonetheless provide valuable insight into participants’ experiences and what they reported during dosing, even if factors like expectation or natural variation in symptoms might be at play.

A key theme in this study was reports of self-determination. This is consistent with other studies indicating motivation for activities during microdosing in healthy individuals,^27^ associated energy, reductions in fatigue, and increases in motivation for exercise.^22,27,29,42–44^ This determination seemed to reinforce motivation and engagement, contributing to improved mood and therapeutic effects in the trial. According to self-determination theory, fulfilling the psychological needs for autonomy, competence, and relatedness is essential for well-being.^41^ In this context, participants’ increased engagement in psychologically beneficial activities may reflect enhanced autonomy and a sense of competence. Numerous studies have shown that activity engagement enhances positive engagement with the world and encourages beneficial behaviours, which may serve as key mechanisms for mood improvement and reduction in depressive symptoms.^41,45,46,47^ These findings also align with Teixeira, Johnson^48^ who suggest that psychedelics should be able to facilitate competence, autonomy, and relatedness and in turn self-determination, and note instances of spontaneous health behaviour changes with psychedelics.^48,49^ Thus, in the present study, participant reports of increased self-determination while microdosing are important insofar as this could be a key mechanism through which LSD microdosing could help alleviate depressive symptoms.

Participants also reported enhanced connectedness to themselves, others, and the world more broadly. This finding aligns with anecdotal reports of LSD microdosing, which have described heightened self-awareness, connection to one’s emotions, and self-acceptance in those with and without mental health conditions.^17,21^ In addition, anecdotal reports suggest that LSD microdosing may enhance social motivation and aptitude, reduce social anxiety, and increase interpersonal connectedness.^16,17,21,22^ Increased connection to nature and a broader sense of interconnectedness with the world have also been noted in previous anecdotal accounts, particularly among those with mental health conditions.^18,21,23,50^ Furthermore, clinical trials of LSD microdosing in healthy individuals also demonstrate transient increases in connectedness to self, others, and nature.^16,29^ Larger doses of psychedelics have demonstrated pro-sociality through increases in trust, empathy, and a desire to be close with others.^51–53^ In the current study, enhanced connection fostered social closeness and a sense of value and belonging in the world, which appeared to contribute to overall well-being and reduction in depressive symptomology. The reported increases in connectedness align with existing research suggesting increased self-connection/awareness^54^ and social connection^55–57^ are major protective factors from depression. Indeed, depression is often described as a ‘disorder of disconnection’, implying that interventions which foster connection, such as microdosing appears to in this study, may help reduce depressive symptoms.^58^ Moreover, the reduction in depressive symptoms through improved connection likely works together with self-determination, where engaging in meaningful activities boosts social involvement engagement with the world and a renewed sense of self-belonging. Together, these processes appear to contribute to the potential antidepressant effects of microdosing with LSD.

In our study, participants also reported increases in cognitive processing. In particular, better emotional processing, mindfulness, resliance, and focus. These findings support non-clinical data from microdosers suggesting increased mindfulness and self-insight in individuals with and without mental health conditions.^17^ Anecdotal reports also suggest that LSD microdosing increases psychological resilience, flexibility and emotional stability.^23^ In addition, many individuals report that enhancing their ability to cope is a motivation for microdosing.^59^ Previous research also suggests that both macro and microdoses of psychedelics are linked to higher mindfulness levels.^60,61^ In addition, anecdotal research suggests that many individuals microdose to enhance creativity or intelligence,^16^ and report reductions in mind-wandering^6^ and improved attentional focus.^18^ It is important to note that such reports contradict findings from clinical microdosing investigations that have failed to find consistent cognitive, working memory or cognitive improvements in healthy individuals.^27–29,42,43,62^ However, these null findings may be due to a ceiling effect in healthy individuals. Thus, the present study suggests that LSD microdosing might help a clinical population (participants with depression) shift from excessive internal focus and rumination, allowing them to be more present in their lives and gain a broader perspective.

Our findings of improved emotional well-being are also consistent with non-clinical investigations of LSD microdosing in depression that suggest emotional benefits, including enhanced mood^16,19,23,24,63^ and relief from depressive symptoms.^16,21,22^ However, clinical trials of microdosing generally report only small, transient changes in mood, if any changes are observed at all.^27–29,42,62^ Similarly to the aforementioned cognitive/mental clarity outcomes, a ceiling effect may be relevant, where participants were already psychologically well, limiting any potential improvements. In contrast, our findings more closely align with reports from individuals with depression who microdose independently outside of a clinical trial setting.^16,19,24^ Improvements in emotional well-being may also be linked to self-determination, increased connectedness and enhanced cognitive processing (as noted above). These factors are often targeted in mental health interventions to promote better mood and a reduction in depressive symptoms.^50,55,56,64,65^

Participants also noted microdosing with LSD sometimes resulted in unwanted side effects consistent with other microdosing clinical trials, including headaches^28^ and anxiety.^29^ In contrast to other studies noting jitteriness and concentration issues,^8^ these effects were not reported in this sample. In line with previous research on microdosing in healthy individuals, it appears that microdosing does not produce consistent effects for everyone^27,28^ with some studies finding little to no impact in the areas of interest. Much like conventional antidepressants,^14^ it seems LSD microdosing is not universally effective. However, unlike antidepressants, which often cause a broad range of side effects such as emotional numbing, sexual dysfunction and weight gain,^15^ the side effects of LSD microdosing reported here appear to be fewer and milder. These observations underscore the potential advantages of our titration protocol, which not only seeks to reduce adverse effects but also aims to guide individuals into a therapeutic zone for microdosing. Furthermore, the relatively mild side effects observed suggest that the titration approach may have been effective for some individuals in reducing side effects.

### Implications

The findings of this study demonstrate the different experiences of individuals microdosing LSD for their depression in an open-label trial. In particular, they point to the potential of titration protocols in microdosing interventions. In this context, participants’ ability to titrate their dose appeared to help them remain in a subjectively optimal range (or ‘therapeutic zone’), allowing for personalised dosing that avoided both sub-therapeutic levels and doses associated with adverse effects. In this case, if participants experienced adverse effects, they were encouraged to reduce their next dose. This flexibility is likely essential if microdosing is to be implemented in clinical practice, where sensitivity to LSD and therapeutic response might vary considerably across patients.

Secondly, the study design itself, which included encouragement to engage in daily and meaningful activities, may have contributed to participants’ experiences. Our observations suggest a cyclical relationship in which self-determination, connectedness and cognitive processing appeared to reinforce one another and were associated with improved emotional well-being. Including structured activities may have supported this cycle, with participation likely enhancing self-determination, fostering connection, improving cognitive processing, and, in turn, reducing depressive symptoms. Therefore, incorporating activity engagement in future research and clinical applications could help initiate or strengthen this cycle.

Thirdly, the observed differences in the efficacy of the microdosing protocol indicate that it may benefit some individuals but not others. Microdosing is not a universal solution, and expectations should be managed accordingly. Overall, these findings LSD microdosing could provide benefit for those with MDD. However, further research is required to clarify optimal dosing strategies and identify predictors of response.

### Limitations

While this qualitative study provides value to the field. The lack of placebo control presents a significant limitation, meaning any results cannot be attributed solely to microdosing. Reported changes may have been influenced by factors like trial involvement or engagement in study activities.^66–68^ For example, participants were encouraged via the app to engage in mood-enhancing behaviours (e.g. meditation, exercise, or treating themselves) about an hour after dosing. It remains unclear whether increased self-determination resulted from LSD or from these encouraged behaviours or a combination of both. The absence of a placebo also introduces potential for expectancy effects, as participants were aware they were receiving the drug, which may lead to preconceived notions about its effects. This is especially relevant in the psychedelic field, where media hype and the promise of significant outcomes can amplify expectations^69–71^ and possibly moderate outcomes.^23,68^ To mitigate this in future trials, two arms should follow the same protocol, but with one group receiving an active placebo that mimics certain subjective effects, making it harder to discern their condition (i.e. a stimulant such as caffeine or low doses of methylphenidate). Future work should include quantitative assessments of symptom trajectories, enabling systematic evaluation and comparison of improvements or lack thereof across groups.

Another limitation of our study was that the participants, by chance, mostly had moderate to low levels of depression. Thus, the results from this qualitative investigation may or may not apply to those with more severe levels of depression. Subsequent research should include a broader range of depression severities to investigate this further.

## Conclusion

The present work provides in-depth insight into the experience of individuals with MDD who participated in an open-label LSD microdosing trial. Participants reported that microdosing promoted self-determination, enabling them to re-engage with the world, fostering a broad sense of connectedness to themselves, others and their environment, along with enhanced cognitive processing. We posit that these three factors appear to create a multi-directional positive feedback loop, ultimately contributing to improvements in emotional well-being. However, it is important to note that some participants reported side effects, highlighting the need for a titration protocol to minimise adverse effects. Further, a subset of participants noted no symptom change, emphasising that microdosing is not necessarily a panacea for all individuals. Given the open-label design, reported benefits or side effects may reflect expectations or natural variation in depression rather than LSD itself, so no causal link can be inferred. Nonetheless, these findings provide a first step for future placebo-controlled trials.

## Supplemental Material

sj-docx-1-tpp-10.1177_20451253251396253 – Supplemental material for What is it like to microdose LSD for depression? a thematic analysis of participant interviews from an open-label trial Supplemental material, sj-docx-1-tpp-10.1177_20451253251396253 for What is it like to microdose LSD for depression? a thematic analysis of participant interviews from an open-label trial by Carina Joy Donegan, Dimitri Daldegan-Bueno, Rachael L. Sumner, Anna Forsyth, Will Evans, Nicholas R. Hoeh, Frederick Sundram, David Menkes, Suresh Muthukumaraswamy and Lisa Reynolds in Therapeutic Advances in Psychopharmacology

## Declarations

**Ethics approval and consent to participate:** The authors assert that all procedures contributing to this work comply with the ethical standards of the relevant national and institutional committees on human experimentation and with the Helsinki Declaration of 1975, as revised in 2013. Ethics approval was awarded by the University of Auckland Health and Disability Ethics Committee (HDEC) on the 14th of December 2022 (Reference: 13536), regulatory approval by the HRC Standing Committee on Therapeutic Trials (Online reference: 2022/SCOTT/13545; Department of Health reference TT55-0335 (2988)) with permission to prescribe Class A substances approved by The Minister of Health. Participants gave written informed consent prior to undertaking the trial, and consent was orally reconfirmed at each in-person trial visit.

**Consent for publication:** All participants provided written informed consent, allowing the research team to transcribe and analyse and publish their interview data. All participant quotes were anonymised, and any identifying information was removed before completing this manuscript.

**Availability of data and materials:** The authors do not have permission to share data. Access to the final trial dataset is only available to the study investigators and any other relevant regulatory bodies.
