# Supplementary material for: What is it like to microdose LSD for depression? a thematic analysis of participant interviews from an open-label trial
Source: Ther Adv Psychopharmacol. 2025 Dec 4;15:20451253251396253. doi: 10.1177/20451253251396253 (PMC12681616; doi:10.1177/20451253251396253)
Supplement: sj-docx-1-tpp-10.1177_20451253251396253 – Supplemental material for What is it like to microdose LSD for depression? a thematic analysis of participant interviews from an open-label trial [file sj-docx-1-tpp-10.1177_20451253251396253.docx]

**Supplementary Materials**

**LSDDEP1: Semi-structured Interview Guide**

**Time:**Approx. 30 minutes **Purpose:**Qualitative analysis, elicit and record experiential aspects of LSD microdosing **Format:**In person at visit 4 (Measure 1, day 57 +/- 2) and in person at visit 6 (Measure 2, EXT day 57 +/- 2) for LSDDEP 1 and 2. **Data recording and analysis:**interviews are to be recorded using lab microphones, then transcribed with dictation software and analysed using Nvivo analysis software to identify themes in participant experience.

**Introductory Statement**

This is going to be your opportunity to really tell us in more detail how you found being in the trial. There are a few things we want to cover but this is really a space to explore how it’s been for you.

Audio recording

It will be audio-recorded and transcribed so that we can analyse your experience and that of other participants, the interviews content will be confidential, and any quotes will be anonymised if used.

This usually takes around 30 minutes but can be longer or shorter. That's up to you.

Are you okay with that?

Is it alright for me to start recording?

*NB: general prompts to be used throughout the interview to encourage talking and expand on one-word answers (yes/no/sort of etc.) e.g., "in what way? ", "how come? ", "can you tell me a bit more about that?"*

| Begin recording by saying participant code and session: "This is LDA0XX Interview Measure XX." |
| --- |

I just want to open with a short Karakia to set the tone and open up the space for you to share your experience. Is that alright with you?

*Mauri oho*
*Mauri tū*
*Mauri ora*
*ki a tātou*
*Haumi e, Hui e*
*Tāiki e!*

(Life force awaken, Life force stand tall, Life force all wellness, good health for all , Join together, unite, the group is ready to progress for the purpose of coming together)

**Interview Section: Experiences**

A really nice place for us to start is to get a sense for how it has been being on this trial for you...

Make sure to ask about the following, changes in...

- Overall Mental Health (taha hinengaro)
- Depression
- Physical health (taha tinana)
- Energy
- Ability to manage problems
- Feelings about whānau and friends, colleagues
- Social
- Spiritual wellbeing (wairua)
- Thoughts about future
- Habits (food, exercise, self talk)
- Sleep
- Concentration
- Creativity
- Emotions

Have other people noticed any changes in you? What were they?

Now that you have completed an LSD regimen how do you feel about future options for treatments for depression?

Before we move to the next stages are there any other changes you would like to note that we haven't talked about yet?

**Interview Section: Protocol**

Now we are going to move on to the next stage focusing more about your experiences with the protocol, things like... the app, the dosing itself etc.

- Just so we can get an idea if we are doing things the best way. So how did you find the protocol?
- Was anything hard to understand in being part of the trial?
- How was the load? i.e. the number of questionnaires and visits etc
- How was the Garmin?
- How was the app?

Do you have any suggestions for us for future studies? Anything we could have done better?

**Interview Section: Closing**

Now we are coming to the end of the interview, is there anything you would have liked to talk about that we haven't yet? Just going to close with a final few questions:

- What would you tell another person who is considering taking part in this trial?
- If this became available as a treatment, what thoughts would you have about that?
- Finally, if you had to sum it up what was your experience with the study and its effects?

Thank you so much for sharing your experience with me today, I would just like to close with karakia.

*Kia tau kia tātou katoa
Te āio, te aroha me te marutau*
*Tihei Mauri Ora*

(May peace, love, and safety Be upon us all Tihei Mauri Ora)

**Standards for Reporting Qualitative Research (SRQR) Checklist**

O’Brien B.C., Harris, I.B., Beckman, T.J., Reed, D.A., & Cook, D.A. (2014). Standards for reporting qualitative research: a synthesis of recommendations. *Academic Medicine, 89(9)*, 1245-1251.

| **No. Topic** | **Item** | **Present?** |
| --- | --- | --- |
| **Title and abstract** |  |  |
| S1 Title | Concise description of the nature and topic of the study identifying the study as qualitative or indicating the approach (e.g., ethnography, grounded theory) or data collection methods (e.g., interview, focus group) is recommended | ✓ |
| S2 Abstract | Summary of key elements of the study using the abstract format of the intended publication; typically includes objective, methods, results, and conclusions | ✓ |
| **Introduction** |  |  |
| S3 Problem formulation | Description and significance of the problem/phenomenon studied; review of relevant theory and empirical work; problem statement | ✓ |
| S4 Purpose or research question | Purpose of the study and specific objectives or questions | ✓ |
| **Methods** |  |  |
| S5 Qualitative approach and research paradigm | Qualitative approach (e.g., ethnography, grounded theory, case study, phenomenology, narrative research) and guiding theory if appropriate; identifying the research paradigm (e.g., positivist, constructivist/interpretivist) is also recommended | ✓ |
| S6 Researcher characteristics and reflexivity | Researchers’ characteristics that may influence the research, including personal attributes, qualifications/experience, relationship with participants, assumptions, or presuppositions; potential or actual interaction between researchers’ characteristics and the research questions, approach, methods, results, or transferability | ✓ |
| S7 Context | Setting/site and salient contextual factors; rationale^a^ | ✓ |
| S8 Sampling strategy | How and why research participants, documents, or events were selected; criteria for deciding when no further sampling was necessary (e.g., sampling saturation); rationale^a^ | ✓ |
| S9 Ethical issues pertaining to human subjects | Documentation of approval by an appropriate ethics review board and participant consent, or explanation for lack thereof; other confidentiality and data security issues | ✓ |
| S10 Data collection methods | Types of data collected; details of data collection procedures including (as appropriate) start and stop dates of data collection and analysis, iterative process, triangulation of sources/methods, and modification of procedures in response to evolving study findings; rationale^a^ | ✓ |
| S11 Data collection instruments and technologies | Description of instruments (e.g., interview guides, questionnaires) and devices (e.g., audio recorders) used for data collection; if/how the instrument(s) changed over the course of the study | ✓ |
| S12 Units of study | Number and relevant characteristics of participants, documents, or events included in the study; level of participation (could be reported in results) | ✓ |
| S13 Data processing | Methods for processing data prior to and during analysis, including transcription, data entry, data management and security, verification of data integrity, data coding, and anonymization/deidentification of excerpts | ✓ |
| S14 Data analysis | Process by which inferences, themes, etc., were identified and developed, including researchers involved in data analysis; usually references a specific paradigm or approach; rationale^a^ | ✓ |
| S15 Techniques to enhance trustworthiness | Techniques to enhance trustworthiness and credibility of data analysis (e.g., member checking, audit trail, triangulation); rationale^a^ | ✓ |
| **Results/Findings** |  |  |
| S16 Synthesis and interpretation | Main findings (e.g., interpretations, inferences, and themes); might include development of a theory or model, or integration with prior research or theory | ✓ |
| S17 Links to empirical data | Evidence (e.g., quotes, field notes, text excerpts, photographs) to substantiate analytic findings | ✓ |
| **Discussion** |  |  |
| S18 Integration with prior work, implications, transferability, and contribution(s) to the field | Short summary of main findings; explanation of how findings and conclusions connect to, support, elaborate on, or challenge conclusions of earlier scholarship; discussion of scope of application/generalizability; identification of unique contribution(s) to scholarship in a discipline or field | ✓ |
| S19 Limitations | Trustworthiness and limitations of findings | ✓ |
| **Other** |  |  |
| S20 Conflicts of interest | Potential sources of influence or perceived influence on study conduct and conclusions; how these were managed | ✓ |
| S21 Funding | Sources of funding and other support; role of funders in data collection, interpretation, and reporting | ✓ |

^a^The rationale should briefly discuss the justification for choosing that theory, approach, method, or technique rather than other options available, the assumptions and limitations implicit in those choices, and how those choices influence study conclusions and transferability. As appropriate, the rationale for several items might be discussed together.
